# Segmentation-guided photon pooling enables robust single-cell analysis and fast fluorescence lifetime imaging microscopy

**DOI:** 10.1117/1.JBO.31.7.076501

**Published:** 2026-07-02

**Authors:** Kayvan Samimi, Danielle E. Desa, Xiaotian Zhang, Dan L. Pham, Rupsa Datta, Melissa C. Skala

**Affiliations:** a Morgridge Institute for Research, Madison, Wisconsin, United States; b University of Wisconsin, Department of Biomedical Engineering, Madison, Wisconsin, United States

**Keywords:** autofluorescence imaging, photon pooling, decay fitting, lifetime estimation, NADH, fluorescence lifetime imaging microscopy

## Abstract

**Significance:**

Fluorescence lifetime imaging microscopy (FLIM) can probe the metabolic environment of living cells in a label-free and noninvasive manner. However, endogenous fluorophores have low absorption and quantum yields, requiring long integration times to acquire the high photon counts needed for accurate pixel-wise multi-exponential decay fitting.

**Aim:**

A computationally light “region-of-interest” photon pooling technique was used to expedite label-free, single-cell FLIM acquisition and analysis, and its accuracy was compared with standard fitting techniques.

**Approach:**

We first characterized the accuracy and precision of “region-of-interest” photon pooling using known fluorescence standards and tested its ability to recover fluorescence lifetimes of single cells and large regions of interest with low photon budgets.

**Results:**

Single-cell metabolic information was accurately extracted from scanning periods as low as 1 s, and large FLIM mosaics were acquired 15 times faster than was possible with conventional pixel-level analysis. Lifetimes extracted using photon pooling were comparable to standard measurements requiring much longer integration times. The technique was also applied to measure fluorescence lifetimes in highly dynamic live samples.

**Conclusions:**

“Region-of-interest” (ROI) photon pooling extracts fluorescence lifetimes from live, dynamic samples with low photon budgets, expediting image acquisition while preserving cell-level or ROI-level lifetime information while sacrificing intra-ROI spatial resolution. The technique is computationally light, does not require machine learning algorithms, and can be integrated with commonly used analysis software and file types.

## 1 Introduction

Fluorescence lifetime imaging microscopy (FLIM) has become a powerful tool for assessing living biological specimens. FLIM is sensitive to the local environment of the fluorophore, including pH, oxygen concentration, temperature, and conformational changes with protein binding.^1^^,^^2^ Autofluorescent metabolic coenzymes, reduced nicotinamide adenine dinucleotide (phosphate) (NAD(P)H) and oxidized flavin adenine dinucleotide (FAD), provide a label-free source of contrast for FLIM^3^ and can be used to study changes in cell metabolism over time in a nondestructive manner.^4^^,^^5^

Measuring lifetime decays from autofluorescent biomolecules presents unique challenges. First, the quantum yield, defined as the ratio of emitted to absorbed photons, of autofluorescent biomolecules (e.g., NAD(P)H) is 30 to 40× lower than that of conventional engineered fluorophores (e.g., green fluorescent protein, tdTomato).^6^^–^^9^ Second, resolving the fluorescence lifetime of autofluorescent biomolecules requires more complex analysis because these molecules exist in multiple binding states. For example, NAD(P)H can exist freely in a cell or bind to over 300 other molecules in various metabolic and biosynthetic reactions.^10^ NAD(P)H can also self-quench when unbound, resulting in a shorter lifetime (∼400  ps) relative to its bound state (∼2 to 5 ns).^9^^,^^11^ More photons (>1000/pixel after spatial binning)^12^ are therefore needed for multiexponential fitting to accurately recover complex autofluorescence lifetimes, often resulting in longer image acquisition times and potential photodamage. Acquisition times on the order of 1 min per autofluorescence FLIM image are common. This restricts the ability of autofluorescence FLIM to capture highly dynamic (∼1  s) biological processes in live cells.^13^

FLIM hardware solutions provide one approach to reduce autofluorescence FLIM acquisition times. Time-correlated single-photon counting (TCSPC) is the most sensitive and photon-efficient time-resolved acquisition technique in low-light settings, providing excellent signal-to-noise ratio (SNR) and timing resolution.^13^^,^^14^ Although most TCSPC FLIM implementations rely on single-point laser scanning systems, parallel excitation can be used to simultaneously illuminate multiple points in the sample and thereby reduce acquisition times.^3^^,^^13^ For example, multifocal FLIM^15^^,^^16^ or light sheet illumination^17^^,^^18^ can be combined with high-speed detectors such as single-photon avalanche diode arrays^19^^–^^21^ to produce sufficient SNR to resolve TCSPC lifetime decays using faster acquisition times than single-point laser scanning methods.^15^^–^^18^

Alternatively, post-processing techniques can be used to compensate for faster acquisition times and the resulting low SNR FLIM images. Lifetime decays are typically fit in a pixel-wise manner using iterative re-convolution with the instrument response function (IRF) through weighted least squares (WLS) or maximum-likelihood estimation (MLE) optimization, which require hundreds of photons per pixel to achieve accurate fits.^3^^,^^13^ Local spatial pixel binning (i.e., aggregation of neighboring pixel decays in a 3×3, 5×5, or larger kernel) is regularly employed in pixel-wise fitting (e.g., SPCImage software) to achieve the required photon counts. Alternative approaches such as global fitting algorithms, which assign "best guesses" to a subset of the lifetime fit parameters based on spatial distributions within a sample, can decrease the number of photons per pixel needed for fitting.^22^^–^^24^ Similarly, Bayesian analysis determines the likelihood function and lifetime from a prior fluorescence decay to establish and then maximize the posterior distribution of parameters using fewer photons per pixel than iterative re-convolution methods.^25^^,^^26^ More recently, “smart binning” techniques based on spatiotemporal correlation between pixels^27^^,^^28^ and deep learning neural network–based algorithms^29^^–^^31^ have been developed to recover lifetimes in images with fewer than a hundred photons per pixel. However, these post-processing techniques usually incur high computational costs or require case-specific pretraining and are therefore not frequently used. In practice, live-cell FLIM is commonly performed using commercial TCSPC electronics that are easily integrated into many imaging systems. Pixel-wise fitting (after local spatial pixel binning) using WLS or MLE^32^ is then typically performed in a graphical user interface prior to image segmentation or region-of-interest (ROI) selection for further statistical analysis.^13^

Here, we developed a simple and computationally cheap technique that enables fast (low-photon) FLIM acquisition in a manner compatible with a widely used acquisition and analysis pipeline without the need for specialized electronics or machine learning algorithms. This method intentionally trades intracellular spatial resolution for improved lifetime fitting performance and is therefore best suited to studies where pixel-level lifetime maps would otherwise be summarized into cell-level or ROI-level features. We first characterize the performance of “region of interest” (ROI)-binned analysis compared with conventional pixel-level analysis using a uniform fluorescence sample, investigating the effects of photon budget on accuracy and precision. We compare the performance of the ROI-binned analysis to the Cramér-Rao lower bound (CRLB) on lifetime estimation variance for the same photon budget range. We then establish the ability of ROI-binned analysis to accurately recover NAD(P)H lifetimes in living cancer cells with low photon budgets. We take advantage of the reduced photon budget requirements of the ROI-binned analysis to capture fast single-cell dynamics in moving neutrophils (∼1  s acquisition per frame providing ∼1.5  photons/pixel or ∼2000  photons/cell) and beating cardiomyocytes (∼1  s/  frame) and then to capture large field of view (FOV) mosaics (1.8×1.8  mm) of HeLa cells with short (1 min) acquisition times (equates to ∼15× larger FOV for the same acquisition time). These results demonstrate a user-friendly and computationally light approach for recovering autofluorescence lifetime parameters in biological samples. This workflow is adaptable for commonly used analysis software and preserves single-cell information without the need for computationally expensive spatiotemporal correlation analysis or complex machine learning methods.

## 2 Materials and Methods

### 2.1 Theoretical Lower Bound of Fluorescence Lifetime Estimation Precision

The CRLB on fluorescence lifetime estimation variance was calculated for the same sample and imaging system parameters as the fluorescent solution experiment (described in Secs. 2.2 and 2.3) using open-source code written by Bouchet et al.^33^ and implemented in MATLAB (MathWorks, Inc., Natick, Massachusetts, United States).

### 2.2 Sample Preparation: Fluorescence Standards and Live-Cell Culture

Reduced nicotinamide adenine dinucleotide (NADH) (Sigma-Aldrich 43420, Burlington, Massachusetts, United States) was dissolved in Tris-buffered saline (diH2O, 50 mM Tris, 150 mM NaCl, pH 7.6) at a concentration of 50  μM to minimize self-quenching (that occurs above 375  μM^34^). 10  μM glucose-6-phosphate dehydrogenase (G6PDH, Sigma-Aldrich G6378) was added immediately prior to imaging to bind NADH in solution and produce multiexponential fluorescence decays (expected protein bound fraction at 10  μM is ∼20%^35^). A single droplet was placed on a No. 1.5 glass-bottomed imaging dish (MatTek Corporation, Ashland, Massachusetts, United States) and imaged using a digital (scan) zoom of 10× to produce a uniformly filled FOV (30×30  μm imaged area) in the center of the scan range, as seen in Fig. 2.

PANC-1 human pancreatic (ATCC CRL-1469) and HeLa human cervical (ATCC CCL-2) adenocarcinoma cells were maintained in high-glucose Dulbecco’s modified Eagle’s medium supplemented with 10% fetal bovine serum (Gibco) and 1% penicillin/streptomycin (Gibco). $3\times 10^{5}\text{ cells}$ were plated on each glass-bottomed dish 24 h prior to imaging and maintained at 37°C and 5% ${\mathrm{CO}}_{2}$.

Primary human neutrophils were isolated from the peripheral blood of a healthy donor using the MACSxpress Whole Blood Neutrophil Isolation Kit (Miltenyi Biotec, Bergisch Gladbach, Germany), following the manufacturer’s instructions within 1 h of blood draw. Erythrocyte depletion (Miltenyi Biotec) was performed according to the manufacturer’s instructions after isolation. The cells were resuspended in Roswell Park Memorial Institute (RPMI) 1640 Medium (Gibco, Waltham, Massachusetts, United States) supplemented with 10% adult bovine serum, and 1% penicillin-streptomycin and kept at 37°C under 5% ${\mathrm{CO}}_{2}$. For imaging, neutrophils were plated at 200,000 cells per 50  μL of medium on 35 mm glass-bottom dishes (MatTek) precoated with Cell-Tak (Corning). Blood draws were performed following protocols approved by the Institutional Review Board of the University of Wisconsin–Madison (2018–0103), and informed consent was obtained from all donors.

Cardiomyocytes were differentiated from WTC-11 human-induced pluripotent stem cells following an established method.^36^ Cardiac progenitor cells were plated on Matrigel-coated glass-bottomed plates (Ibidi) on day 5 post-differentiation and maintained in RPMI (Gibco) with B27 + insulin (LifeTechnologies, Carlsbad, California, United States) at 37°C and 5% ${\mathrm{CO}}_{2}$.^37^ Images were acquired^38^ in fully differentiated, beating cardiomyocytes (19 days post-differentiation). Cells were incubated with Mitotracker Orange CMTM (25 nM, Invitrogen, Carlsbad, California) at 37°C and 5% ${\mathrm{CO}}_{2}$ for 15 min, rinsed three times, and immediately imaged in fresh culture medium.

### 2.3 Fluorescence Lifetime Imaging

Imaging was performed on an Ultima two-photon imaging system (Bruker, Billerica, Massachusetts, United States) with an ultrafast tunable laser source (80 MHz, 100 fs pulses; Insight DS+, Spectra Physics, Milpitas, California, United States) coupled to a Nikon Ti-E inverted microscope with TCSPC electronics (SPC-150, Becker & Hickl, and Time Tagger Ultra, Swabian Instruments, Stuttgart, Germany). NAD(P)H was excited at 750 nm and emissions collected using a 440/80  nm bandpass filter (Chroma, Taoyuan City, Taiwan) and GaAsP photomultiplier tube (H7422PA-40, Hamamatsu). Mitotracker Orange (cardiomyocyte mitochondrial imaging) was excited at 750 nm and emission collected using a 590/50  nm bandpass filter. The system instrument response function (IRF, full width at half maximum ∼240  ps) was acquired from the second harmonic-generated signal of urea crystals at 750 nm, captured on the same detector. Samples were illuminated through a 40×/1.15  NA objective (Nikon, Shinagawa-ku, Tokyo, Japan), with ∼6  mW laser power at the sample. PANC-1 and HeLa cells were imaged with a digital zoom of 1 (300  μm FOV), neutrophils with a digital zoom of 2 (150  μm FOV), and cardiomyocytes with a digital zoom of 2.5 (120  μm FOV). Fluorescence decays (256-time bins) were acquired across 512×512-pixel images with a pixel dwell time of 4.8  μs. FLIM frames (i.e., a single pass of the galvanometer scanner over the FOV, ∼1.5  s/frame) were saved to disk individually. The required number of consecutive FLIM frames was combined to emulate FLIM images with total integration times from 1 to 120 s for the NADH solution and PANC-1 cells.

### 2.4 Mosaic FLIM

The Atlas Imaging feature of the Prairie View (Bruker, Billerica, Massachusetts, United States) microscope control software was used to program a serpentine pattern of microscope stage positions covering a large area of the cell plate (1.8×1.8  mm). FLIM images were acquired sequentially and saved separately. The images were fused in post-processing using the BigStitcher^39^ plugin in Fiji.^40^

### 2.5 Image Denoising and ROI Segmentation

NAD(P)H intensity images were denoised using convolutional neural networks (CNN) where necessary for accurate single-cell segmentation. CNN denoising was used only when raw intensity images were too photon-limited for reliable segmentation. The denoised images were used only for mask generation and visualization, not for lifetime fitting. In the case of neutrophil images, a generative accumulation of photons (GAP) network^41^ designed for mitigating shot noise in photon counting datasets was trained on ∼500 noisy images of neutrophils (without the need for any noise-free ground-truth images) and used to denoise the experimental sequence of 1 s images of neutrophils before and after activation with phorbol 12-myristate 13-acetate (PMA) treatment. The pretrained CNN included in Cellpose3^42^ was used to denoise the HeLa cells mosaic intensity images. It should be noted that denoising is performed only on the intensity images for the sole purpose of generating cell masks and therefore does not change the lifetime data used in the fitting analyses. We may also use the denoised intensity image for visualization of the color-coded lifetime image.

The uniform fluorescence standard (NADH solution) images were segmented into a grid of square ROIs with 10, 20, 40, 60, 80, or 100 pixels (width) for analysis. Automated cell segmentation was performed on NAD(P)H intensity images using Cellpose^43^^,^^44^ (model “cyto2” for PANC-1; custom models for neutrophils; model “cyto3” for HeLa cells). The napari viewer was used to manually segment cardiomyocytes and perform any necessary mask corrections in the other cell types.

### 2.6 Pixel-Wise Multiexponential Fitting

Pixel-wise multiexponential fitting was performed using SPCImage v 8.8 (Becker & Hickl, Berlin, Germany). Two-component decays were calculated at each pixel by the following equation: $I(t)=\alpha_{1}e^{-t/\tau_{1}}+\alpha_{2}e^{-t/\tau_{2}}+C$, where $\alpha_{1}$ and $\alpha_{2}$ are the amplitudes, $\tau_{1}$ and $\tau_{2}$ are the decay lifetimes of the fast and slow decay components, respectively. C is the constant nondecaying background light amplitude. A binning factor of 1 (3×3  pixels) was used for all analyses.^3^ Both WLS and MLE iterative fitting and re-convolution with the IRF were used to fit multiexponential fluorescence decays.^13^

**Fig. 1:**
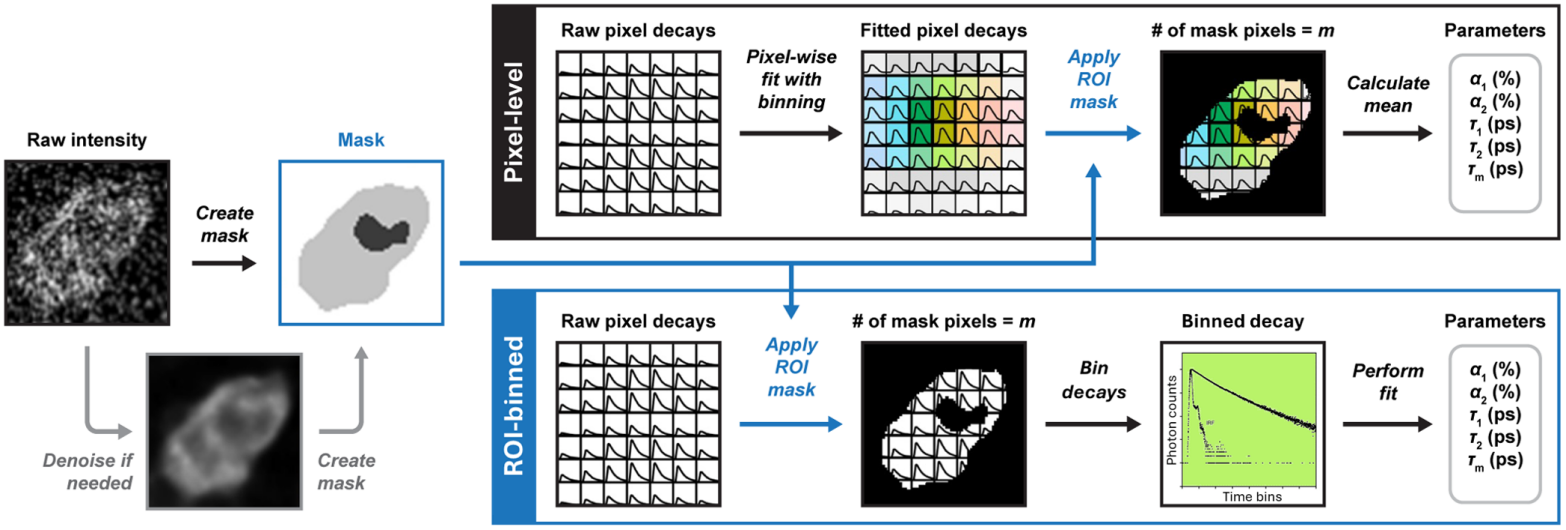
Analysis pipeline for single-cell fluorescence lifetime extraction. Pixel-level: Raw fluorescence decays can be fitted to a multiexponential model at each pixel prior to cell segmentation. Single region/cell masks are applied, and average lifetime fit parameters are then calculated for each ROI. ROI-binned: Binary cell masks defining cell boundaries are applied to the raw FLIM images, and a single fluorescence decay is calculated from the sum of all pixel decays contained in the ROI. Lifetime parameters are then extracted at the single-ROI level by fitting this decay.

Segmentation was performed as described in Sec. 2.5, and lifetime fit parameters ($\alpha_{1},\tau_{1},\alpha_{2},\tau_{2}$) exported from SPCImage were averaged over all pixels within each ROI using “cell analysis tools,”^45^ a custom Python library that calculates the average of pixel-wise lifetime fit parameters over the pixels of each ROI (Fig. 1).

### 2.7 ROI-Binned Multiexponential Fitting

Segmentation was performed on the uniform fluorescent sample or live-cell images as described in Sec. 2.5 and masks were applied to the raw FLIM image files. For each mask object (e.g., single cell or ROI), the decays from all object pixels were combined to produce a decay with high photon count (Fig. 1). This aggregate “ROI-binned” decay was then identically assigned to all pixels of the object. This “new” FLIM image file was subsequently loaded and fitted in SPCImage using both WLS and MLE methods, as described in Sec. 2.6. The bin factor was set to zero because these modified images are effectively pre-binned. The lifetime fit parameters were extracted using “cell analysis tools.”^45^’ ROI pooling sums decays from pixels within the same biological ROI, so the same bi-exponential decay model used for pixel-wise NAD(P)H fitting was applied to the pooled decay. This assumes that the ROI contains the same dominant lifetime species, with pooling primarily increasing photon counts rather than changing the underlying model.

## 3 Results

### 3.1 NADH Solution: Characterizing Performance with a Controlled Photon Budget

**Fig. 2:**
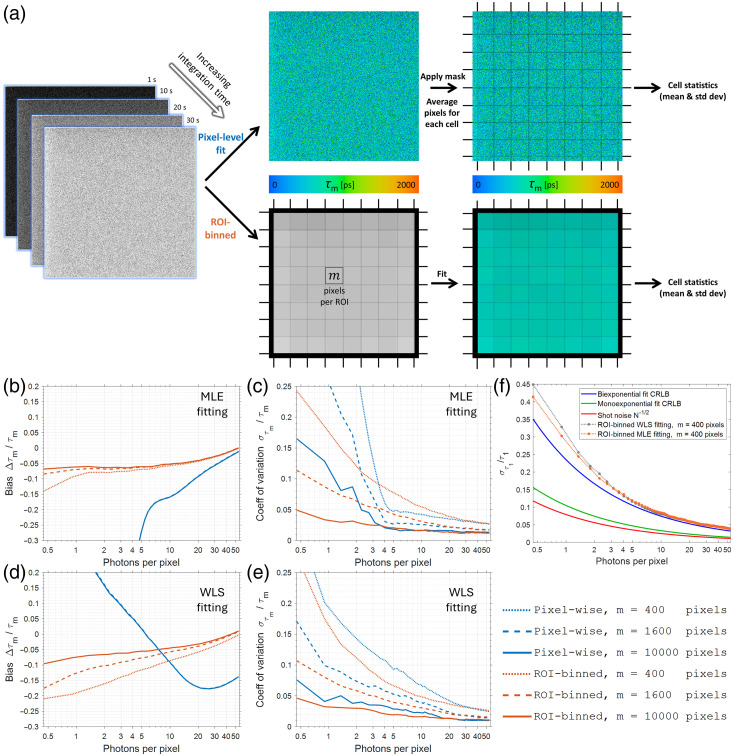
Uniform NADH solution phantom characterizes performance of fitting methods. (a) A uniform solution of 50  μM NADH+10  μM G6PDH was imaged as a sequence of 120 single FLIM frames (∼1  s each) that were combined in post-processing to yield integration times ranging from 1 to 120 s (equivalent to 0.45 to 54  photons/  pixel). The resulting FLIM images were then divided into a grid of squares simulating single cells (several grid sizes) and analyzed using pixel-wise or ROI-binned fitting methods in SPCImage. In the pixel-wise pipeline (top row), the left lifetime image preserves pixel-level lifetime estimates, whereas the right image overlays the grid ROIs used to average pixel-wise fit parameters for the cell-level statistics in panels (b)–(e). In the ROI-binned pipeline (bottom row), the m pixel decays within each grid cell are summed to produce a high-count decay in the left image, which is then fitted to produce the grid cell lifetime estimate in the right image. (b), (c) The normalized bias and standard deviation of the estimates using MLE fitting. (d), (e) The normalized bias and standard deviation of the estimates using WLS fitting. (f) The normalized standard deviation of the $\tau_{1}$ lifetime estimates from the ROI-binned MLE and WLS fitting analyses approach to the theoretical CRLB calculated using the “ground-truth” lifetimes of the NADH solution: Background photons=10%, $\tau_{1}=336\;\mathrm{ps}$, $\tau_{2}=2371\;\mathrm{ps}$, $\alpha_{1}=85\%$, $\alpha_{2}=15\%$.

We first characterized the performance of the pixel-level and ROI-binned methods (Fig. 1) using a uniform sample (NADH solution) and controlled photon count per pixel [Fig. 2(a)]. The laser power was adjusted to attain a count rate of $∼1.2\times 10^{5}\text{ photons}/s$, ensuring adequate SNR while avoiding photon pile-up artifacts. We first calculated the ground-truth multiexponential lifetime parameters for the bound NADH solution (glucose-6-phosphate dehydrogenase added) from the aggregation of 120 single FLIM frames. At this integration time, the average image pixel decay contains 54 photons, which is typical of autofluorescence images of cells. With sufficient pixel binning, these photon numbers can provide accurate and repeatable lifetime estimates. Both pixel-level (with a binning factor >1) and ROI-binned analysis pipelines, using either MLE or WLS fitting methods, converge on a biexponential decay profile with these parameters: Background photons=10%, $\tau_{1}=336\;\mathrm{ps}$, $\tau_{2}=2371\;\mathrm{ps}$, $\alpha_{1}=85\%$, $\alpha_{2}=15\%$, where background photons refer to the nondecaying ambient light intensity, also known as offset. The “ground-truth” mean fluorescence lifetime (defined as $\tau_{m}=(\alpha_{1}\tau_{1}+\alpha_{2}\tau_{2})/(\alpha_{1}+\alpha_{2})$) of NADH in solution was found to be 641 ps.

Given this ground truth, we then normalized and presented the bias and coefficient of variation (COV) across grid “cells” for lifetime estimates by each analysis pipeline [Figs. 2(b)–2(e)]. The independent variables in this experiment are photons per pixel on horizontal axes, and cell size, presented as the number of pixels per square grid cell.

The performance of ROI-binned analysis (orange curves) was compared with pixel-wise fitting with a bin factor of 1 (blue curves) using the MLE [Figs. 2(b) and 2(c)] or the WLS [Figs. 2(d) and 2(e)] methods. ROI-binned analysis results in a smaller negative bias compared with pixel-wise analysis using MLE. This indicates that pixel-wise fitting tends to underestimate lifetime, particularly at very low photons per pixel (≤1), a regime where ROI-binned fitting can provide usable estimates. The residual WLS bias at the highest pixel photon counts reflects the limited photon budget available to each locally binned pixel decay for bi-exponential fitting. Using ROI pooling, the larger pooled photon budget reduces this dependence on fitting algorithm efficiency, such that both MLE and WLS fits of ROI-binned data have vanishing biases as photon budget increases. Similarly, the COV for the ROI-binned WLS fitting pipeline is lower than its pixel-wise equivalent over the entire range of photons per pixel. However, the COV for the ROI-binned MLE fitting pipeline is only lower than the pixel-wise MLE fitting pipeline at the higher end of photons per pixel (where the pixel-wise fitting bias has shrunk to <5%).

Given the previous results, we compared the standard deviation of our unbiased lifetime estimators (MLE, WLS) to the theoretical minimum value given by the Cramér-Rao lower bound^33^ [Fig. 2(f)]. The three CRLB curves correspond to increasing model complexity: a background-free mono-exponential shot-noise limit (i.e., $1/\sqrt{N}$), a mono-exponential model with nonzero background (i.e., three estimated parameters: lifetime $\tau_{1}$, its amplitude $\alpha_{1}$, and offset C), and a bi-exponential model with nonzero background (i.e., five estimated parameters: lifetime $\tau_{1}$, its amplitude $\alpha_{1}$, lifetime $\tau_{2}$, its amplitude $\alpha_{2}$, and offset C). The bi-exponential-with-background bound is the relevant comparison for the NADH solution fits in this experiment. The theoretical curves were calculated using the ground-truth solution lifetime parameters and a cell size of m=400  pixels. The ROI-binned standard deviation approaches the CRLB over the entire photon budget range, with the ROI-binned MLE fits providing a smaller standard deviation compared with the WLS fits. In summary, ROI-binned fitting lowers both estimation bias and COV compared with pixel-wise fitting by combining decay photons belonging to the same cell object prior to performing the fit, thus alleviating the effects of photon shot noise more effectively.

### 3.2 Comparison of Pipelines in Live-Cell Images at Varying Integration Times

Having characterized the performance of each pipeline in a uniform solution, we next performed a real-world comparison of the pipelines in FLIM images of cancer cells. In addition to pixel-wise and ROI-binned fitting pipelines, we also processed the images using CASPI,^27^ a computational pre-processing algorithm that collaboratively uses local and nonlocal spatio-temporal correlations between pixel decays to recover decay profiles in low-light regimes. At the cost of additional computation, the CASPI algorithm effectively spatially and temporally denoises the 3D FLIM data cube while preserving pixel-level spatial resolution.

**Fig. 3:**
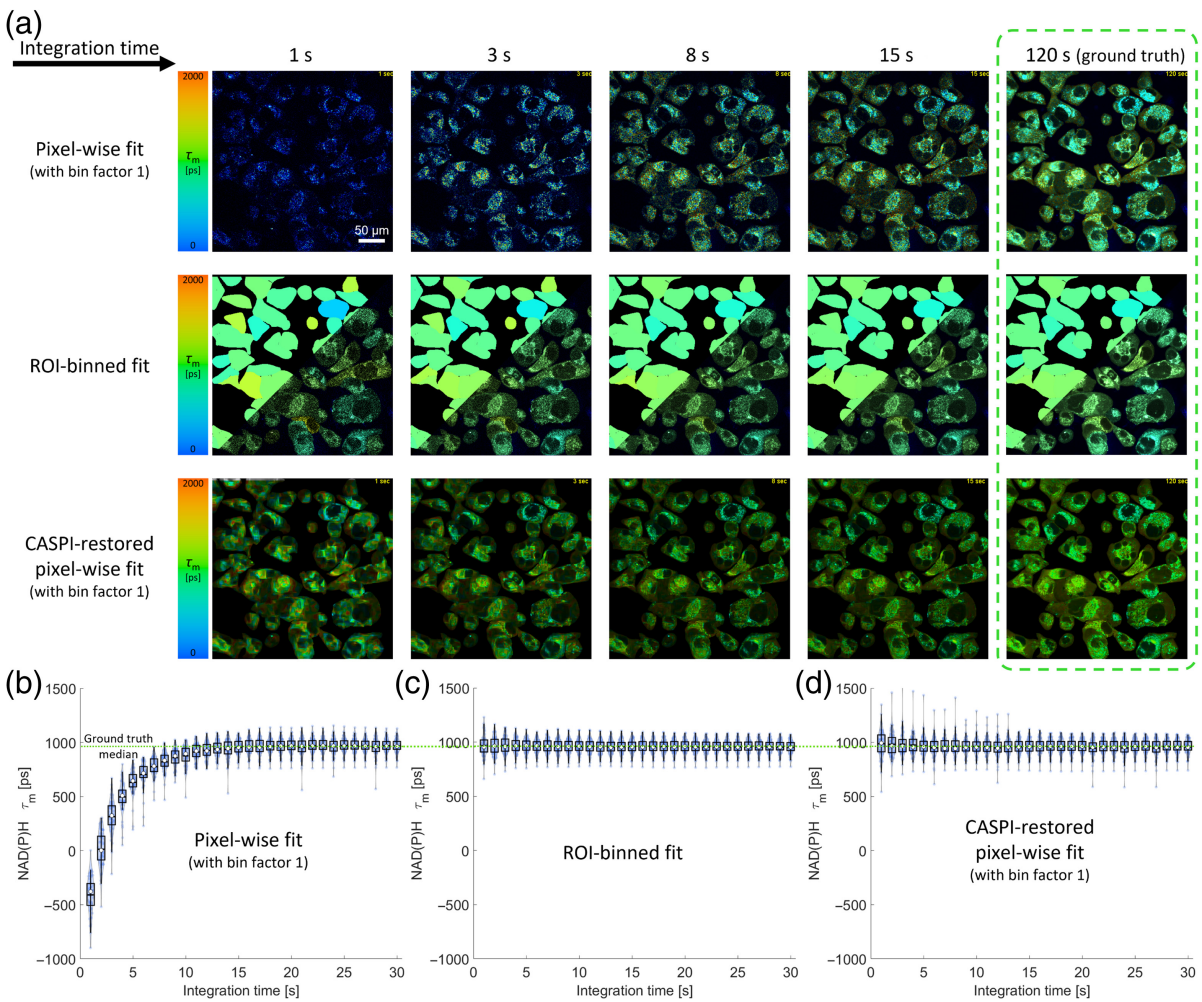
ROI-binned analysis produces valid autofluorescence lifetime estimates within 1 s of integration. Adherent PANC-1 cells were imaged as a real-world test case. (a) Representative FLIM images with increasing integration times when analyzed using the conventional pixel-wise MLE fitting, ROI-binned MLE fitting, or CASPI restoration followed by pixel-wise MLE fitting, all using SPCImage software. Violin plots of the single-cell mean lifetime distribution with increasing integration times are given for panel (b) conventional pixel-wise MLE fitting, (c) ROI-binned MLE fitting, and (d) CASPI-restored pixel-wise MLE fitting. The ROI-binned and CASPI-restored FLIM images provide valid lifetime estimates from the first second of integration, but conventional pixel-wise fitting only converges to the true lifetimes after about 15 s of integration (Video 1, MP4, 5.27 MB [URI: https://doi.org/10.1117/1.JBO.31.7.076501.s1]).

A single FOV of PANC-1 cells was imaged using multiphoton FLIM with varying integration times (1 to 120 s) generated by aggregating increasing numbers of single frames as before. The photon counts were maintained at a constant rate ($2\times 10^{5}\text{ photons}/s$). The FLIM images were analyzed one of three ways: conventional pixel-wise MLE fitting with a binning factor of 1 (3×3  pixels), ROI-binned MLE fitting, or pixel-wise MLE fitting with a binning factor of 1 after pre-processing using the CASPI algorithm^27^ before extracting single-cell lifetime parameters (Fig. 3). The ground-truth NAD(P)H mean lifetime ($\tau_{m}$) was calculated using pixel-wise MLE fitting of 120 s acquisition, and mean lifetimes converged to the true value (<5% bias) after ∼15  s.

Pixel-wise MLE fitting produces invalid negative lifetime estimates in the first 3 s of integration due to very low photons per pixel [Fig. 3(b)]. By contrast, ROI-binned MLE analysis produces estimates within 5% bias with just 1 s integration [Fig. 3(c)]. The resulting lifetime images are presented with flat lifetime color-coding alone or superimposed on pixel-level intensity for visualization [Fig. 3(a)]. Like ROI-binned MLE analysis, CASPI restoration before fit analysis results in pixel-level estimates within 5% bias with just 1 s integration [Fig. 3(d)]. Video 1 presents FLIM images produced by these pipelines for the first 15 s of integration.

### 3.3 Application: Dynamic Imaging of Primary Neutrophils

**Fig. 4:**
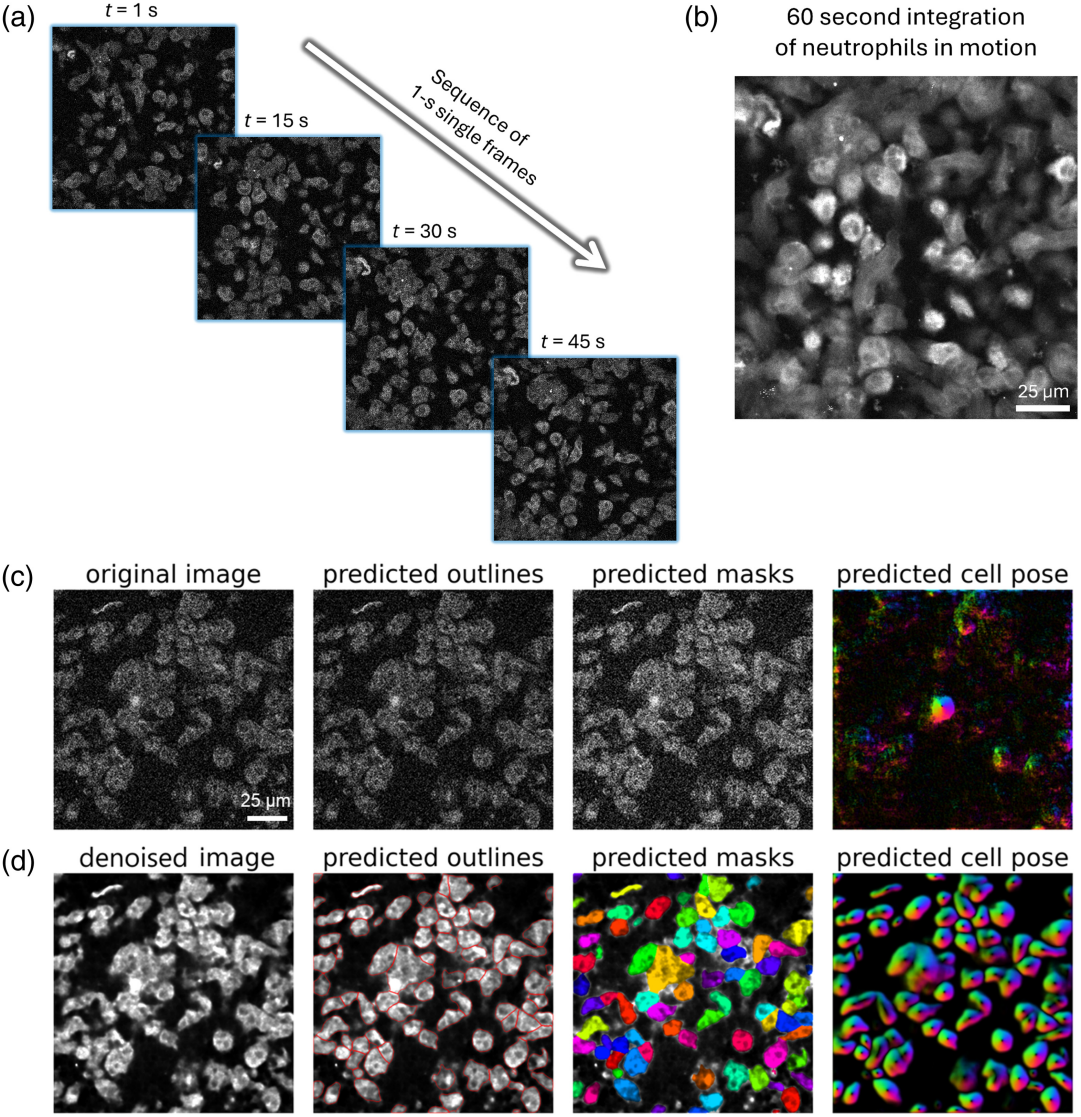
CNN-based denoising enables single-cell segmentation and lifetime estimation for fast FLIM imaging. (a) Representative intensity images of migrating neutrophils captured as a series of single-frame FLIM images. (b) Combining all frames for a conventional 60 s integration time results in a blurred image that cannot be segmented, and FLIM analysis would suffer from crosstalk between cells. FLIM images would ideally be analyzed on a per-frame basis. However, cell segmentation in Cellpose fails (c) due to the heavy influence of shot noise in single-frame intensity images. (d) Denoising single-frame images using a GAP neural network enables successful single-cell segmentation necessary for ROI-binned analysis.

Neutrophils are highly dynamic and fast-responding innate immune cells. Conventional FLIM with minute-long integration times fails to capture rapid metabolic shifts in neutrophils, and the movement of neutrophils leads to blurred images [Fig. 4(b)]. To test the feasibility of ROI-binned analysis for capturing these dynamic processes, we applied the pipeline to single-frame (∼1  s each) FLIM images of these neutrophils immediately before and after the addition of a chemical activator, phorbol 12-myristate 13-acetate (PMA). To this end, single-frame cell masks are required to identify pixels corresponding to individual cells. However, attempting to segment these single-frame intensity images in Cellpose^44^ leads to failure due to the effects of shot noise at these low photon counts [Fig. 4(c)]. We trained and applied a GAP denoising neural network^41^ to mitigate the shot noise in these single-frame intensity images. As a result of this denoising, Cellpose segmentation of single cells in single-frame images is now successfully performed [Fig. 4(d)].

**Fig. 5:**
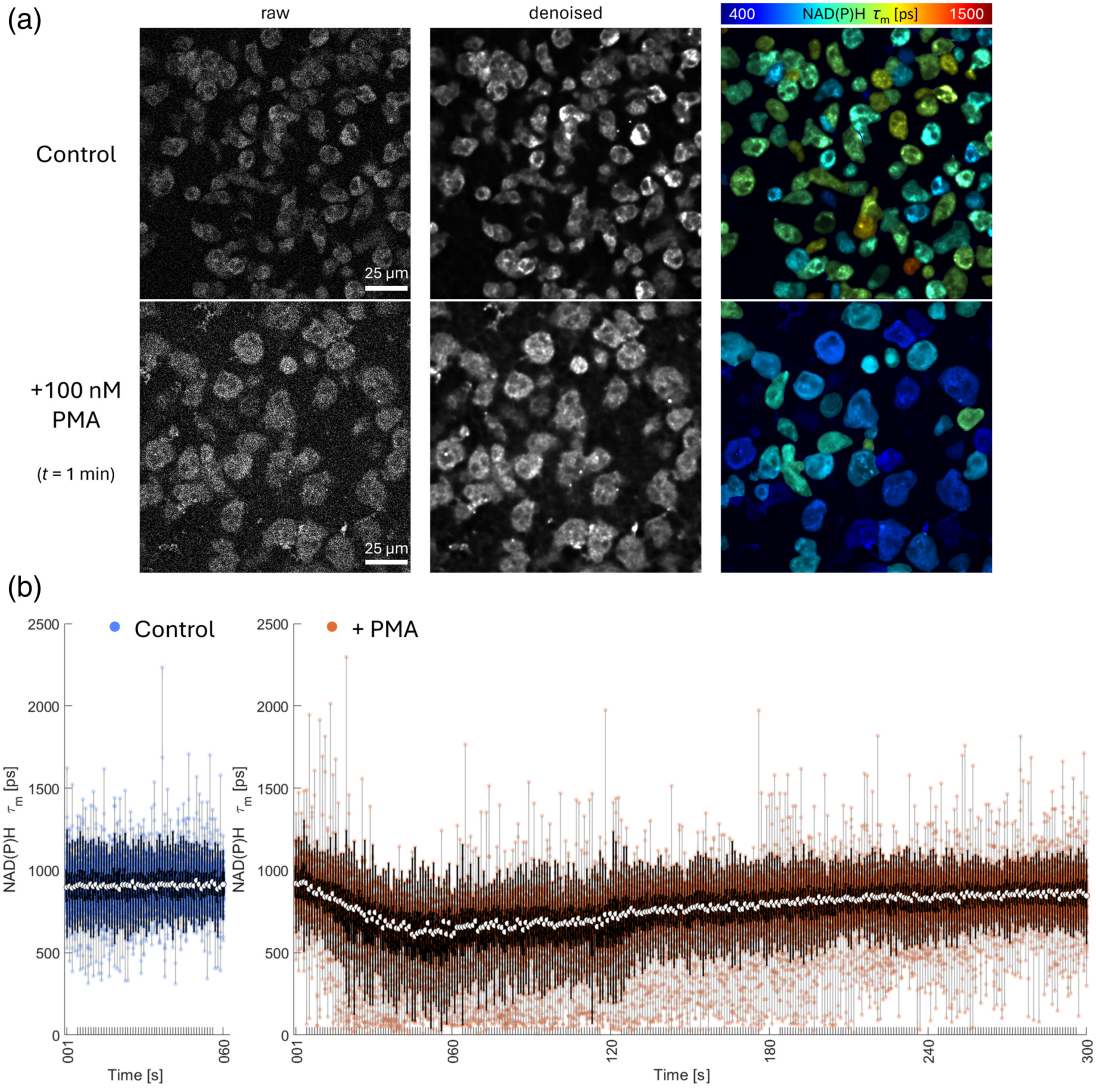
ROI-binned FLIM imaging captures fast metabolic dynamics in activated migrating neutrophils. (a) Representative raw intensity, denoised intensity, and ROI-summed mean lifetime superimposed on denoised intensity images of neutrophils before and 1 min after treatment with PMA. (b) Time series boxplots of single-cell NAD(P)H lifetime distributions in the same FOV for 60 s before (left, control, blue) and 5 min after (right, +PMA, red) treatment with PMA in 1-s intervals. This 1-s resolution reveals fast metabolic dynamics with the mean lifetime dropping for 1 min after PMA treatment and gradually recovering over the next few minutes. White dots represent the median value, dark box shows the interquartile range, and whiskers indicate the ±1.5× interquartile range (Video 2, MP4, 9.53 MB [URI: https://doi.org/10.1117/1.JBO.31.7.076501.s2]).

Representative raw, denoised, and ROI-binned mean lifetime images of the neutrophils before and 1 min after activation with PMA are shown in Fig. 5(a). The single-cell mean lifetime distributions over the course of 6 min (1 min before and 5 min after PMA activation) are plotted in 1 s intervals (Fig. 5(b)). NAD(P)H mean lifetimes are stable over 1 min in control neutrophils. In comparison, upon treatment with PMA, the mean lifetime drops within the first minute and subsequently begins to slowly recover over the next few minutes (Video 2). Beyond population lifetime trends presented in Fig. 5(b), single-cell tracking and morphological analysis was performed using the TrackMate^46^ plugin in ImageJ. The results are presented as a ranked heatmap in Fig. S1 in the Supplementary Material where NAD(P)H mean lifetime, cell area, circularity, and motility are presented as scaled z-values over the 300-s imaging time course. It can be generally observed that the cells that were tracked immediately after PMA treatment (at t=0  s) and present with the early drop in NAD(P)H lifetime (in the first 120 s) are more motile than the cells that were tracked later (after 120 s post treatment). It is also observed that jumps in instantaneous motility coincide with decreased circularity of the cells. These rapid changes would not be visible with the much longer acquisition time typically required for autofluorescence FLIM, showcasing a clear use for this analysis method.

### 3.4 Application: Mitochondrial Imaging in Beating Cardiomyocytes

We next used the ROI-binning method for recovering NAD(P)H lifetimes in conjunction with organelle staining to visualize dynamic, subcellular lifetime information in beating cardiomyocytes. These cells are highly metabolically active and known to have extensive mitochondrial networks. A sequence of 60 single-frame fluorescence lifetime images was acquired in two spectral channels, capturing NAD(P)H autofluorescence and MitoTracker Orange simultaneously. An Otsu thresholding of the MitoTracker intensity channel was used to generate mitochondrial network masks, which were then combined with single whole-cell masks to produce single-cell mitochondrial network ROIs. The NAD(P)H fluorescence lifetime decays were ROI-binned and analyzed within these single-cell mitochondrial networks. Mean lifetime values for mitochondria were color-coded and presented, as seen in Video 3. Mitochondrial mean fluorescence lifetime changes rapidly throughout different phases of the beat cycle and is heterogeneous across cells. This example demonstrates that the ROI-binning pipeline can be combined with subcellular organelle labels to perform fast autofluorescence FLIM of specific cell structures.

### 3.5 Application: Mosaic Imaging of Large FOV

**Fig. 6:**
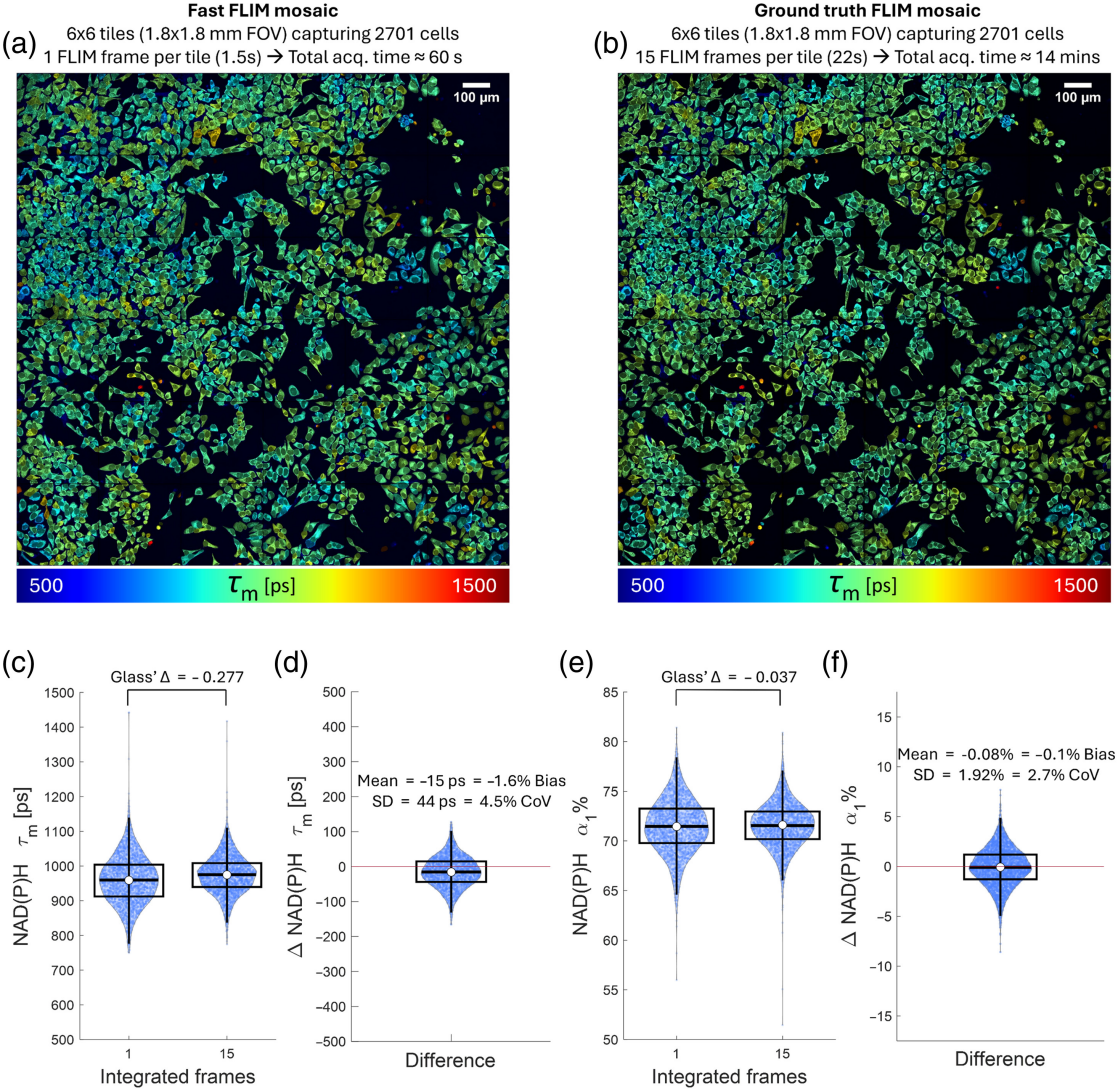
ROI-binned analysis enables fast large-FOV mosaic FLIM imaging. (a) 6×6 fast FLIM mosaic using a single frame (∼1.5  s/frame) at each tile position captured 2701 HeLa cells within 1 min. By contrast, acquiring the (b) “ground-truth” FLIM mosaic by accumulating 15 frames at each tile position requires 14 min of total integration time. Both mosaics were analyzed using ROI-binned MLE fitting, and the color-coded lifetime values are superimposed on the corresponding denoised intensity image. (c) Distribution of single-cell NAD(P)H mean lifetimes for single-frame and 15-frame integration. (d) Distribution of mean lifetime difference between the two FLIM images, showing an average bias of −1.6%. (e) Distribution of single-cell free fraction of NAD(P)H for single-frame and 15-frame integration. (f) Distribution of free fraction difference between the two FLIM images showing an average bias of −0.1%. Box plot shows the interquartile range and whiskers extend from the box to 1.5× the interquartile range. The white dot shows the median, and the horizontal line shows the mean. Glass’s Deltas were calculated with respect to 15-frame integrated image as the effect size (Video 3, MP4, 9.52 MB [URI: https://doi.org/10.1117/1.JBO.31.7.076501.s3]).

Autofluorescence FLIM of large FOVs is often time-consuming owing to long integration time requirements. ROI-binned lifetime analysis would considerably increase the image acquisition speed in such cases, enabling stitching of many FOVs to cover a large area. Mosaic images consisting of 6×6 FOV tiles ($1.8\times 1.8\;{\mathrm{mm}}^{2}$) were acquired of HeLa cells using a serpentine pattern of microscope stage locations. A ground-truth mosaic was acquired by integrating the FLIM image at each stage position for 15 passes of the scanning galvos (∼22  s), requiring a total acquisition time of about 14 min. A fast mosaic was then acquired by taking a single FLIM frame (∼1.5  s) at each position for a total acquisition time <1  min. The images were fused using the BigStitcher^39^ plugin in Fiji^40^ and segmented in Cellpose3 using the built-in pretrained one-click denoising neural network.^42^ Both datasets were analyzed using ROI-binned MLE fitting, and color-coded lifetime values were superimposed on denoised intensity images as shown in Figs. 6(a) and 6(b). We compared the single-cell NAD(P)H mean lifetime [$\tau_{m}$, Fig. 6(c)] and free fraction [$\alpha_{1}$, Fig. 6(e)] measured in the ground truth (15 frames integrated) and fast FLIM (single-frame) mosaics and present the difference between the lifetime variables measured from each mosaic [Figs. 6(d) and 6(f)]. The FLIM images are visually similar, and the quantified difference suggests that taking a single FLIM frame of each FOV only results in a −1.6% mean lifetime ($\tau_{m}$) bias or a −0.1% proportion of short lifetime ($\alpha_{1}$) bias in extracted single-cell lifetime parameters while reducing total imaging time by a factor of ∼14.

## 4 Discussion and Conclusion

We presented an ROI-binned lifetime fitting method as a photon-efficient alternative to pixel-wise fitting that recovers accurate single-cell lifetime parameters. Along with a considerable decrease in FLIM acquisition times, this approach results in improved accuracy (lower bias) and precision (lower standard deviation) of lifetime estimates compared with pixel-wise fitting with the same photons per pixel. We experimentally quantified this method using a uniform NADH solution for a complex decay fitting scenario with realistic photon counts and instrument effects. This approach is a natural extension of spatial pixel binning, which is already a common practice for increasing photon counts during pixel-wise fitting analysis. However, we aggregated all pixels in the same cell or ROI in the image rather than blindly grouping neighboring pixels (3×3, 5×5, etc.,), as is common with typical spatial binning. We further demonstrated these advantages in a real-world live-cell imaging case by analyzing FLIM images from PANC-1 cells acquired over a range of photons per pixel. ROI-binned fitting still requires a biologically meaningful mask, but it is less sensitive to small boundary errors compared with unweighted averaging of pixel-wise lifetime estimates because the pooled decay is dominated by photons from brighter cellular pixels.

The increased photons per decay afforded by ROI-binning enabled us to image both faster single-cell dynamics and larger mosaic FOVs compared with pixel-wise fitting. We demonstrated the ability to track fast metabolic dynamics in motile, activated neutrophils and beating cardiomyocytes with FLIM frame rates of about 1 frame per second. Machine learning advancements in image denoising under shot-noise conditions (which is the dominant noise in TCSPC FLIM) enabled successful segmentation of single cells in noisy fast FLIM images which, in turn, enabled ROI-binning for successful single-cell lifetime estimation. Denoising is not an inherent requirement of ROI-binned fitting. It becomes useful only when the photon budget is pushed low enough that shot noise prevents reliable segmentation, as in the 1 s integration neutrophil example. Applying a pretrained denoising model is computationally light relative to model training, and generalist models such as “one-click denoising” in Cellpose3 may be sufficient for many samples, whereas custom training may be needed for unusual morphologies such as multilobed nuclei in neutrophils.

This ROI-binned fitting approach is hardware agnostic and can be applied to TCSPC images from any multiphoton or confocal system to improve estimation accuracy, increase acquisition speed, reduce excitation light dose and photodamage to cells, and reduce computational cost (as fewer iterative fitting operations are needed compared with pixel-wise fitting). The ROI can be defined as any desired group of pixels for either whole-cell binning (e.g., PANC-1, HeLa, neutrophils) or specific subcellular regions (e.g., mitochondrial network in cardiomyocytes). Thus, ROI can be defined as cytoplasm, nucleus, mitochondria, or other organelles with or without the assistance of live-cell labels. Irrespective of the ROI, binning pixels together prior to re-convolution fitting results in improved fitting performance compared with pixel-wise fitting followed by averaging. We have incorporated ROI-binned fitting into an open-source interactive end-to-end analysis software for single-cell fluorescence lifetime data, FLIM Playground.^47^

ROI-binned fitting is not intended for studies where intra-ROI heterogeneity or pixel-level spatial patterns are the primary biological readout. In situations where preserving pixel-level spatial resolution is desired, global fitting, smart binning algorithms such a sCASPI, or other machine learning algorithms that restore pixel decays under low-photon conditions or directly recover lifetime parameters in fit-free fashion can be used.^23^^,^^27^^,^^28^^,^^48^^–^^50^ However, these techniques either have increased computational cost or require the algorithm to be pre-trained for the sample of interest. For fit-free phasor analysis, the phasor of an ROI-pooled decay is mathematically equivalent to the intensity-weighted average of the pixel phasors within that ROI, so ROI pooling can also be interpreted as a photon-weighted ROI-level phasor summary.

In conclusion, ROI-binned analysis is a photon-efficient approach to estimate single-cell fluorescence lifetime parameters that, depending on the user priorities, can improve estimation accuracy and precision, or increase imaging speed by a factor of 15 or more. ROI-binned analysis enables autofluorescence FLIM of fast dynamics that would otherwise not be achievable using laser-scanning FLIM, all while lowering the computational cost of single-cell analysis.

## Supplementary Material

10.1117/1.JBO.31.7.076501.s01

10.1117/1.JBO.31.7.076501.s1

10.1117/1.JBO.31.7.076501.s2

10.1117/1.JBO.31.7.076501.s3

## Data Availability

The source code is available upon request and will be released as open source upon publication. An example dataset of PANC-1 cells and basic ROI-binning source code are available at https://github.com/skalalab/ROI_binning.
