# Supplementary material for: Segmentation-guided photon pooling enables robust single-cell analysis and fast fluorescence lifetime imaging microscopy
Source: J Biomed Opt. 2026 Jul 2;31(7):076501. doi: 10.1117/1.JBO.31.7.076501 (PMC13325633; doi:10.1117/1.JBO.31.7.076501)
Supplement: Supplementary file 1 [file JBO_031_076501_SD001.pdf]

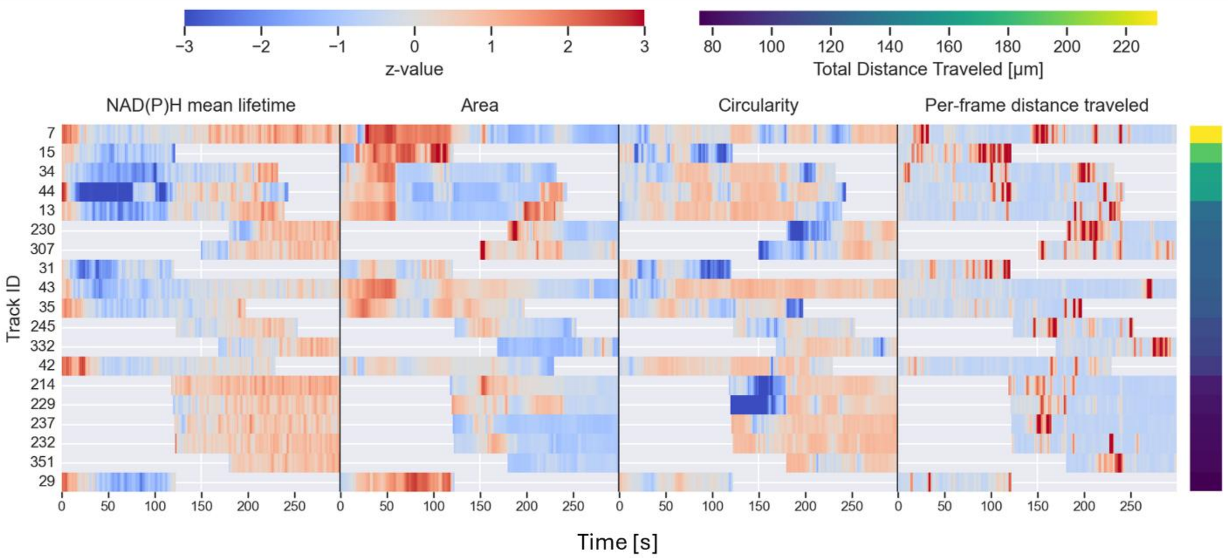

Fig. S1. ROI-binned FLIM enables paired single-cell tracking and autofluorescence lifetime estimation in activated neutrophils. Single-cell tracking and morphological analysis was performed using TrackMate plugin in ImageJ. NAD(P)H mean lifetime, cell area, circularity, and motility (per-frame distance traveled) are presented for each track as scaled z-values over the 300-second imaging time course. Only cells that were continuously tracked for a minimum of 120 s are presented. Empty timepoints (where cells have exited or not yet entered the frame) are displayed as a gray background with a white line through it. The rows are ordered by the total distance traveled (highest to lowest). It can be generally observed that the cells that were tracked immediately after PMA treatment (at $t = 0$ s) and present with the early drop in NAD(P)H lifetime (in the first 120 s) are more motile than the cells that were tracked later (after 120 s post treatment). It is also observed that jumps in instantaneous motility coincide with decreased circularity of the cells. This example highlights the potential for monitoring dynamic cellular behaviors (e.g., cell cycle and division, migration, chemotaxis, phagocytosis) unlocked by fast FLIM using ROI-binned analysis.
